# A Novel Affective Analysis System Modeling Method Integrating Affective Cognitive Model and Bi-LSTM Neural Network

**DOI:** 10.1155/2022/1856496

**Published:** 2022-10-07

**Authors:** Zhuqing Yang, Liya Zhou, Zhengjun Jing

**Affiliations:** ^1^Jiangsu Vocational College of Information Technology, Wuxi, Jiangsu 214153, China; ^2^Changshu Binjiang Vocational and Technical School, Changshu, Jiangsu 215512, China; ^3^Jiangsu University of Technology, Changzhou, Jiangsu 213001, China

## Abstract

The severity of mental health issues among college students has increased over the past few years, having a significant negative impact on not only their academic performance but also on their families and even society as a whole. Therefore, one of the pressing issues facing college administrators right now is finding a method that is both scientific and useful for determining the mental health of college students. In pace with the advancement of Internet technology, the Internet has become an important communication channel for contemporary college students. As one of the main forces in the huge Internet population, college students are at the stage of growing knowledge and being most enthusiastic about new things, and they like to express their opinions and views on study life and social issues and are brave to express their emotions. These subjective text data often contain some affective tendencies and psychological characteristics of college students, and it is beneficial to dig out their affective tendencies to further understand what they think and expect and to grasp their mental health as early as possible. In order to address the issue of assessing the mental health of college students, this study makes an effort to use public opinion data from the university network and suggests a college student sentiment analysis model based on the OCC affective cognitive model and Bi-LSTM neural network. In order to label three different types of positive, negative, and neutral sentiment on the microblog text of college network public opinion, we first design a sentiment rule system based on the OCC affective cognition elicitation mechanism. In order to effectively and automatically identify the sentiment state of college students in the network public opinion, this study uses a Bi-LSTM neural network to classify the preprocessed college network public opinion data. Finally, this study performs comparison experiments to confirm the validity of the Bi-LSTM neural network sentiment recognition algorithm and the accuracy of the OCC sentiment rule labeling system. The findings show that the college student sentiment recognition effect of the model is significantly enhanced when the OCC sentiment rule system is used to label the college network public opinion data set as opposed to the naturally labeled data set. In contrast to SVM and other classification models like CNN and LSTM, the Bi-LSTM neural network-based classification model achieves more satisfactory classification results in the recognition of college opinion sentiment.

## 1. Introduction

In the modern era, issues related to mental health have emerged as an increasingly prevalent public concern associated with significant risks to society [1]. College students, particularly in recent years and especially as a result of the influence of COVID-19, are being subjected to an increasing amount of pressure in the forms of higher education, employment, and competition, all of which contribute to the emotional problems and mental health problems that students experience. College students frequently struggle with a variety of mental health issues, including anxiety, depression, inferiority complex, and interpersonal sensitivity. What's more, a significant number of them have entertained the idea of ending their own lives [2]. There is no doubt that this will have serious adverse effects not only on individuals but also on families and even on society as a whole. If the emotional tendencies and psychological state of the students can be detected early on, the school will be able to provide timely and specific assistance to students who are struggling, which will reduce the amount of damage caused. As a consequence of this, it is extremely valuable to discover an efficient method to identify college students who have issues related to their mental health. People's use of the Internet as a medium for the transmission of information and communication is becoming increasingly widespread as the Internet continues to gain in popularity. College students make up a sizeable portion of the population of people who use the Internet in China. Due to the anonymity and equality offered by microblogging platforms, college and university students are increasingly turning to them as a means of conveying their feelings, elaborating on their positions, and articulating their requirements. Adolescents and college students are especially susceptible to the influence of Internet public opinion because they make up the largest segment of Internet users. This has a negative impact on the students' emotional tendencies as well as their mental health. Only by mining and analyzing the sentiment tendencies of college students in college network public opinion can we understand college students' mental health status in a timely manner and provide targeted treatment and prevention [3]. This is because only by doing so can we grasp college students' sentiment tendency in an all-encompassing manner. As a consequence of this, the analysis of the sentiment of college students in network public opinion needs to be strengthened in order to guarantee the psychological well-being of college students and to preserve the harmonious and stable growth of colleges and universities.

However, due to the large number of subjective texts that are uploaded to the network platform, relying solely on traditional artificial means to analyze these texts will not only require a significant amount of manual labor and a significant amount of time, but it will also significantly reduce the accuracy of recognition. The theory of machine learning is constantly evolving, which has led to an increase in the number of academics who are applying machine learning algorithms to the study of sentiment recognition of university online public opinion. These scholars have found that their efforts have been fruitful [4, 5]. An opinion evolution system model based on complex agent networks was constructed by the literature [6] in order to analyze the influence of various factors within the model on the development of public opinion. This model was constructed in accordance with the characteristics and evolution rules of university public opinion generation. After that, simulation experiments are used to demonstrate the model's viability for use in actual situations. Literature [7] addressed the issue of sparse features in microblog text by employing a labeled LDA model to model microblog text. As a result, the semantic information contained within the text was enriched, and the text's ability to be classified was enhanced. In addition to this, it makes use of technology that aligns words in order to train the translation model, after which it applies this trained model to the university network public opinion analysis. Literature [8] performs an analysis of the characteristics of the text utilizing statistical technology in the form of a word cloud in order to obtain the high-frequency words that were used in user comments. The next step is for them to narrow down the scope of high-frequency words and teach a naive Bayesian classifier in order to finish the text's classification of emotions. At long last, a display of the evolution map of public opinion in colleges and universities as it has developed within the context of microblogs is presented. However, the affective analysis technique that utilizes machine learning depends on the corpus domain, and the contextual knowledge of the context cannot be effectively used, and its classification performance is also affected to some extent. Then, the deep learning techniques improve this problem. It can automatically learn text affective information from a large number of samples and automatically perform feature representation, which provides new ideas for analysis of university opinion and classification of college students' sentiment. Literature [9] makes use of the extensive data set of IMDB film reviews and organizes its textual data into categories that are either positive or negative. After that, based on the data that have been preprocessed, they use a technology called Word2Vec to express the text, and after that, they use an LSTM neural network to analyze the sentiment of the film review text. The results of the test demonstrate the viability and effectiveness of the LSTM model for the analysis of the emotions contained within film reviews.

In light of the information presented above, the authors of this study devise a model for sentiment analysis using an OCC model and a Bi-LSTM neural network, approaching the problem from the perspective of affective cognition theory and deep learning technology. They then use this model to investigate the opinions expressed by college students participating in university online public opinion. The following are some of the novelties that emerged from our research: (1) at the current stage of research on the analysis of the sentiments of online public opinion, the sentiment categories of data sets frequently rely on manual prior labeling, and there is a lack of clear rules or systems. This study contributes to the research by incorporating the traditional OCC affective cognition evaluation model. As a result, the research on the recognition of sentiments in online public opinion has become more standardized. A standardized sentiment rule system is constructed based on the OCC affective cognition elicitation mechanism. In order to construct a dataset, three different types of positive, negative, and neutral sentiment annotations are applied to the crawled microblog texts in university online public opinion. (2) In this study, the characteristics of short texts of university online opinion are combined, and the Bi-LSTM sentiment analysis algorithm is used to train and obtain a sentiment recognition model for college students. The study was conducted by combining the characteristics of university online opinion short texts. The new model is not only capable of resolving the data sparsity problem that plagues traditional machine learning, but it can also improve recognition accuracy by making effective use of feature sequences of textual contextual information. (3) In the final part of this research project, comparison experiments are carried out in order to validate the correctness of the OCC sentiment rule labeling system as well as the scientific merit of the Bi-LSTM neural network sentiment classification algorithm. The experimental results prove that, compared with the natural annotation method, the method based on the OCC sentiment rule system greatly improves the effect of the model on college students' sentiment recognition. What's more, the classification model on the basis of Bi-LSTM neural network achieves more desirable classification results in university opinion sentiment recognition compared with the classification models such as SVM and other deep learning networks like CNN and LSTM. As a result, the utilization of deep learning technology to investigate the feelings held by college students in the context of university-based online public opinion is of utmost significance. It is able to identify the emotional state of college students participating in online public opinion in an effective and automated manner, which makes the monitoring and management of unexpected public opinion much simpler. Concurrently, school teachers are in a position to provide timely attention and regulation in response to the pessimistic sentiment of college students.

## 2. Related Basic Theory

### 2.1. Sentiment Classification

**Figure 1:**
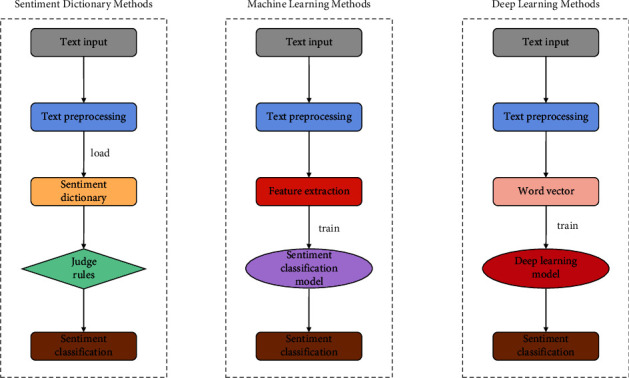
Flow of sentiment classification of three representative methods.

The research on sentiment classification based on online public opinion has become a hot topic as a direct result of the meteoric rise in the amount of data that is stored online as well as the rising demand for the monitoring and management of online public opinion. As a distinct task within the realm of text classification, sentiment analysis shares some parallels with text classification but also highlights some key distinctions. The former focuses on subjective factors as the research object, while the latter centers on objective content as its primary concern. The fundamental task of sentiment classification is to classify texts into two or more types, positive or negative, based on the emotional information that they contain. This task is accomplished by analyzing the texts' content and looking for patterns of positive or negative sentiment. In its most basic form, it can be understood as a categorization of the attitudes and opinions expressed by the publisher [10], a process that is also known as opinion mining in some circles. Researchers have done a significant amount of work in the field of sentiment classification research and have proposed a few research methods that are representative of the field. From these methods, one can derive three more general categories: sentiment dictionary methods [11], machine learning methods [12], and deep learning methods [13].  Figure 1 depicts the processes that each of the three approaches uses to classify the respondents' feelings.

In light of what has been seen in Figure 1, one can reach the following conclusions: the same is that the first step of these three methods is to preprocess the text. That is, data cleaning, word segmentation, and deactivation of Chinese text. The difference lies in the fact that the methods that are based on a sentiment dictionary make use of a previously constructed sentiment dictionary in order to annotate each sentiment symbol in the text, and finally, the formula is designed to calculate the sentiment tendency value of the full text. This is where the distinction lies. Selecting and extracting feature items from the text is the central step of the machine learning methods. These feature items are then used to train a text sentiment classification model. In the final step, the trained model is evaluated for its accuracy in classification using a test set through which it is first put through its training. In contrast, the deep learning methods encode the word vector after preprocessing the text, then use deep neural networks for feature extraction, and finally calculate the probability of each sentiment class by using the softmax function to output the sentiment class. These methods are referred to collectively as “deep learning.” Because the methods are based on a variety of different principles, the practical applications of each of them have their own unique set of benefits and drawbacks. The methods for classifying emotions that are based on sentiment dictionaries are straightforward and easy to put into practice. The construction of a dictionary, on the other hand, necessitates specialized domain knowledge, which results in the methods having poor universality and a limited capacity for generalization. The intelligence of sentiment classification is improved by machine learning methods, but because these methods rely on the corpus domain, contextual knowledge of the context cannot be effectively used, and the classification performance is also impacted to some degree. On the other hand, the methods of sentiment classification that are based on deep learning have a high rate of accuracy and can be applied in a wide variety of contexts. When compared to more conventional machine learning models, it is capable of automatically extracting features on its own. As a consequence of this, the method of sentiment classification based on deep learning is utilized throughout the study.

### 2.2. Text Representation

Text is an unstructured or semistructured form of data organization, which means that computers are unable to directly process it [14]. Text consists of a collection of characters that have been arranged in a certain order. Therefore, if we want to automate the processing of natural language with the assistance of computers, we will first need to convert text into structured data, also known as text representation. This step is necessary because we cannot automate the processing of natural language without it. The representation of text can, in general, be broken down into two categories: discrete representation and distributed representation.

The method of discrete representation known as one-hot coding is common [15]. It is a statistical-based processing method. It regards a word as a symbol, and the dimension of the word vector is the size of the whole dictionary. For each word in the vocabulary, set its corresponding position to 1 and the rest to 0. This method, despite being straightforward and user-friendly, suffers from two major drawbacks, which are as follows: first, because each word in the dictionary is represented by a high-dimensional vector, it is simple to create a dimensional catastrophe when the dictionary is large. This is because each word has its own high-dimensional vector. Second, due to the fact that every pair of words is independent of the other, this technique is unable to capture semantic information, which can easily result in the problem known as the “semantic gap.”

**Figure 2:**
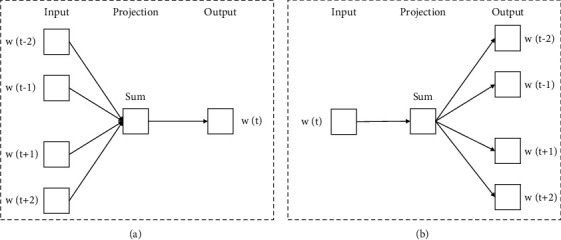
Structure of CBOW and Skip-gram model. (a) CBOW model structure. (b) Skip-gram model structure.

Word2Vec is a method of representative distributed representation [16] that Google developed based on the conventional one-hot model and improved upon it. It is capable of mapping features from a high-dimensional space to a low-dimensional space, thereby resolving the issue of dimensional disaster caused by sparse data in the one-hot model and mining the semantic relationships between words. Word2Vec is essentially a straightforward model of a neural network. Following the completion of the training phase, the text content will be converted into a vector with *K* dimensions. In addition, the similarity of vectors can be utilized in order to convey the similarity of text semantics. Word2Vec performs well in both the classification of text and the classification of sentiment. The one-hot vector serves as the input for the Word2Vec model. After that, the vector is passed on to the neural network model for training. The parameters of the neural network model are continuously adjusted, and the weight matrix is modified as it is being trained in order to obtain distributed vectors. Word2Vec primarily incorporates two distinct variants of the CBOW and Skip-gram models.  Figure 2 presents an illustration of the structure diagrams for both of these models.

When the structure diagrams of the two models shown in Figure 2 are compared and analyzed, we find that CBOW is able to predict the target words given knowledge of the contexts in which the words are found. In order to obtain the word vector associated with the context, it connects the mapping layer directly to the softmax node of the output layer. Additionally, all inputs are projected to the same mapping layer. It does not take into account the word order of the context in which the target word is being used. In contrast to the CBOW theory, the Skip-gram model makes use of the context's target words to make predictions about it. Because of this, the word vector for each word in the context contains the position information that corresponds to that word [17]. However, it leads to a longer training time for the Skip-gram model than the CBOW model. The latter is suitable for training larger datasets, and the former is suitable for a smaller amount of data, and it will make the word vectors more accurate. For the purpose of text vector training in this paper, we make use of the Skip-gram algorithm.

## 3. Evaluation Model Based on Bi-LSTM Neural Network

**Figure 3:**
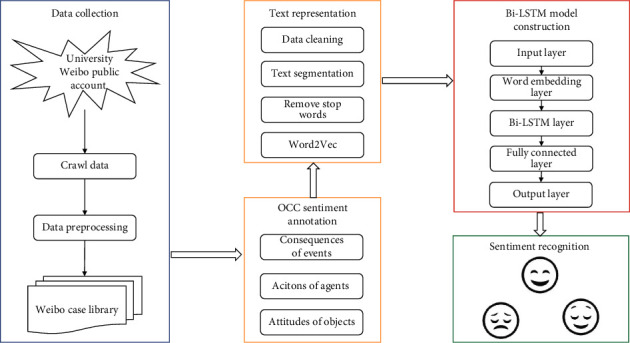
Sentiment analysis model of college students based on OCC model and Bi-LSTM neural network.

From the perspectives of affective cognition and deep learning, this research creates a model for recognizing the emotions of college students by employing the OCC model and the Bi LSTM neural network. These two models are used in conjunction with one another. The real feelings that are implied behind students' text modality data can be mined with the assistance of this model, and the students' psychological shifts over the course of a certain amount of time can be taken into comprehensive consideration in order to achieve a rapid and accurate identification of students' sentiment tendencies. The fundamental structure of the model is shown in Figure 3.

The primary components of the sentiment recognition model developed as a result of this research are shown graphically in Figure 3. (1) The gathering of data: the primary purpose of this section is to implement the sorting, filtering, and cleaning of the data that was gathered by crawlers in order to get the data ready for the subsequent construction of the model. (2) A reasonable sentiment rule system has been established on the basis of the OCC affective cognition model in order to label the text data of online public opinion with three types of positive, negative, and neutral emotions. These emotions are as follows: (3) following the preprocessing step, the text data are still unstructured or semistructured data, which means that computers are unable to recognize them directly. Because of this, we have decided to incorporate the Skip-gram model into the Word2Vec method for the purpose of text vector training. (4) At the end of the process, the Bi-LSTM model is used as a classifier. The final sentiment recognition model is obtained after adjusting the hyperparameters through training, and the positive and negative feelings of college students are evaluated.

### 3.1. Sentiment Annotation Based on OCC Model

When it comes to expressing one's views in the context of human interaction, the use of emotion is an essential component. It is necessary for people to investigate and express their emotions using the sentiment model. The OCC model is the most classical affective cognitive model in cognitive psychology. It is also a psychological sentiment model that is widely used in today's society [18]. It provides a classification scheme for sentiment and provides a scientifically referable basis for standardizing the sentiment labeling system of online public opinion. When it comes to modeling sentiment, the OCC model is the most prominent example. It provides a classification scheme for sentiment as well as a scientifically referable basis for standardizing the sentiment labeling system of online public opinion. As a result of this, the focus of our research is on implementing the OCC model for sentiment annotation of online opinion texts. This allows us to take into account the closed-loop principle and finer levels of sentiment granularity. Its high computability lays the groundwork for investigating the sentiment of university-based online opinion information texts, and its mechanism of affective cognitive elicitation is also an important support for investigating the factors that contribute to the formation of sentiment.

**Figure 4:**
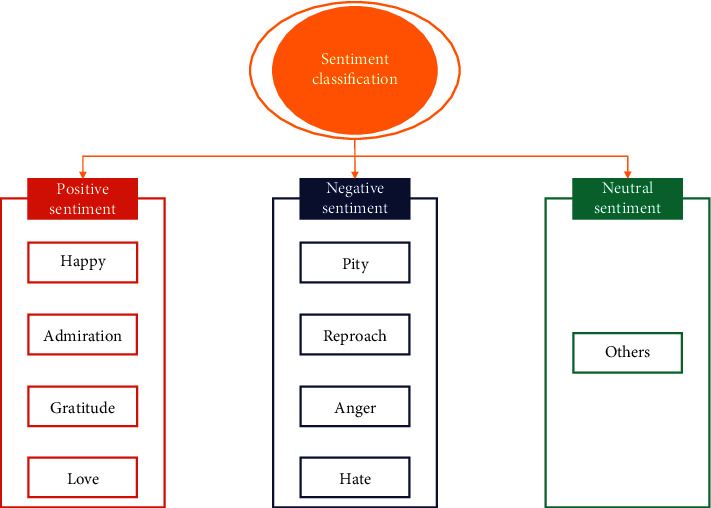
Sentiment types mapping based on OCC model.

The basic sentiment types of college students' online public opinion are determined to be, after screening and combining with the actual situation, the following eight basic emotions: happy, pity, admiration, reproach, gratitude, anger, love, and hate. These are the emotions that have been adopted as the basic sentiment types. It is not necessary to place an excessive amount of emphasis on particular negative sentiment, however, because the research is more inclined to explore the sentiment tendencies in the online public opinion of college students. As a result, it divides the eight OCC basic sentiment types of online public opinion into three distinct categories: positive, negative, and neutral. That is to say, four different types of positive sentiment, such as happiness, admiration, gratitude, and love, are mapped onto the positive sentiment category, whereas four different types of negative sentiment, such as pity, reproach, anger, and hate, are mapped onto the negative sentiment category. As can be seen in Figure 4, the comment text that does not fit into any of these eight categories of sentiment is considered to have a neutral level of sentiment.

A 9-dimensional sentiment space is constructed in this paper, as shown in Figure 4, and a sentiment variable is assigned to each web text, as shown in formula (1).

(1) $$\begin{array}{c} S=\left\{s_{happy},s_{pity},s_{a\;dm\;iration},s_{reproach},s_{gratitu\;de},s_{anger},s_{love},s_{hate},s_{other}\right\}\text{.} \end{array}$$

In formula (1), *s*[0, 1] denotes the value of each dimension of sentiment.

Then, according to the OCC model mapping of the web text, three different formulas for expressing sentiment, as in formulas (2)–(4).

(2) $$\begin{array}{c} S\left(Positive\right)=S\left(happy\right)\cup S\left(a\;dm\;iration\right)\cup S\left(gratitu\;de\right)\cup S\left(love\right)\text{,} \end{array}$$

(3) $$\begin{array}{c} S\left(N\text{egative}\right)=S\left(pity\right)\cup S\left(reproach\right)\cup S\left(anger\right)\cup S\left(hate\right)\text{,} \end{array}$$

(4) $$\begin{array}{c} S\left(Neutral\right)=1-S\left(positive\right)-S\left(negative\right)\text{.} \end{array}$$

### 3.2. Sentiment Recognition Based on Bi-LSTM Model

Textual information is necessary for humans to communicate their feelings and thoughts to one another, and it also plays a significant role in the external representation of a person's mental conditions. It is essential, according to the consensus of opinion expressed online, to accurately determine the state of a college student's mental health by analyzing the mental condition of the student and the sentiment expressed in any content that they post [19]. Even though text is frequently organized as sequential data, the tendency of text can be mined for sentiment effectively based on semantic comprehension if we are able to capture information about the contextual setting of sentences.

This research develops an online opinion sentiment rule that, as was mentioned in Section 3.1, is able to accurately identify the sentiment categories that are present in online opinion texts. This rule is based on the affective cognitive model that was developed by OCC. On the other hand, in today's world, the number of online opinion texts that we need to perform sentiment recognition is in the tens of thousands, hundreds of thousands, or even millions or hundreds of millions. This is because the Internet is filled with people sharing their thoughts. The effectiveness of sentiment classification will be poor if we only manually annotate each web opinion microblog document using the OCC model. Researchers now have a viable option for dealing with large amounts of data, thanks to the rapid development of deep learning. Because of this, the research uses a Bi-LSTM neural network to develop a sentiment recognition model for online public opinion. This is done so that the researchers can quickly and effectively identify the sentiment tendency of college students.

**Figure 5:**
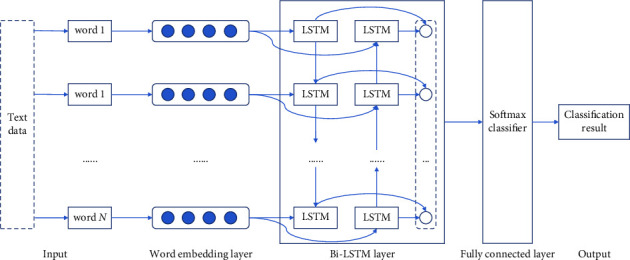
Sentiment analysis model based on Bi-LSTM neural network.

The long short-term memory (LSTM) network is well-known for its distinct gating structure and memory units, which can help avoid problems associated with gradient disappearance and long-term reliance [20]. However, when it learns the features of the text sequence, the information can only propagate in one direction and cannot make good use of the text context information. When applied to the processing of text data, the Bi-LSTM method has the ability to obtain feature sequences that contain text context information. In addition to the information that is unique to it, it includes the information that is associated with the entirety of the text data. Therefore, it contributes to the text's increased capacity for differentiation [21]. Because of this, our research proposes a three-layer neural network architecture consisting of a word embedding layer, a Bi-LSTM layer, and a full connectivity layer that spans the contextual interval to learn the sentiment information implied by the sentences. This will allow for a comprehensive analysis of the semantics of sayings as well as the acquisition of useful representations of sentiment feature representations.  Figure 5 presents a visual representation of the model's flow for recognizing sentiment.

This study employs the Skip-gram model to train the word vector of the text in the word embedding layer because the microblog text exhibits the characteristics of colloquialism and short and succinct expression. As a result, the word vector of the text in this layer is able to more accurately and appropriately represent the text vector.

Following that, two LSTM networks with different timing are connected to the same output in the Bi-LSTM layer [22]. In this situation, the forward LSTM is utilized to extract the text's top-level information, and the backward LSTM is used to get the text's bottom-level information. Assume that *r*_*t*_, *f*_*t*_, *b*_*t*_ stand for the text feature representation at time *t*, the forward hidden state at time *t*, and the backward hidden state at time *t*, respectively*. r*_*t*_ needs to be computed by integrating *f*_*t*_ and *b*_*t*_ in a spliced manner. And *f*_*t*_ is calculated from the input *s*_*t*_ at time *t* and the hidden state information at time *t* − 1. *b*_*t*_ is calculated from the input *s*_*t*_ at time *t* and the hidden state information at time *t* + 1. The details are shown in formulas (5)–(7).

(5) $$\begin{array}{c} r_{t}=f_{t}⊕b_{t}\text{,} \end{array}$$

(6) $$\begin{array}{c} f_{t}=\delta \left(U\times s_{t}+V\times f_{t-1}+\beta \right)\text{,} \end{array}$$

(7) $$\begin{array}{c} b_{t}=f\left(U^{\prime }\times s_{t}+V^{\prime }\times f_{t+1}+\beta^{\prime }\right)\text{,} \end{array}$$

where ⊕ denotes the integration of both in a spliced manner. *δ* denotes the LSTM nonlinear function, *U*, *V*, *U*′, and *V*′ indicate the weights of the function, respectively. *β* and *β*′ represent the bias of the function.

Last but not least, the fully connected layer takes as its input the text feature representation that was obtained from the layer before it, classifies it through the use of a softmax function, and then outputs the computed sentiment tendency values.

## 4. Experimental Testing and Analysis

In order to test the efficacy and reliability of the model designed in this paper in identifying the sentiment of college students' online public opinion, the sentiment analysis model based on OCC and Bi-LSTM neural network is compared with other representative sentiment analysis models, such as SVM, CNN, and LSTM, respectively. The purpose of this comparison is to test the effectiveness and reliability of the model designed in this paper.

### 4.1. Experimental Data

The students at a university located in Jiangsu Province are going to serve as the subjects of this study. In order to carry out the experiment, the comments made by university students on topics that were deemed to be pertinent were crawled from the official microblog account that the university maintains. Both manual annotation and the OCC sentiment rule theory, which was presented in Section 3.1, are utilized as respective foundations for the application of sentiment annotation to the dataset. In this paper, the data that were collected by the crawler are filtered and cleaned up to leave 9600 pieces of data. The members of the project team who understood the OCC sentiment rule system and those who did not understand the OCC model are asked to label the microblog information texts with sentiment classification, respectively, in order to form the online opinion dataset for the university.

### 4.2. Data Processing

In many cases, the quality of the data has an effect on the experimental findings of the sentiment analysis performed on college students. In the meantime, the raw web text data that are crawled from the public microblogs of universities are not suitable for direct utilization in the context of sentiment analysis. Because of this, we need to run these raw data through a series of preprocessing operations such as data cleaning, word separation, and deactivation before we can use them for sentiment analysis of text. These operations include cleaning the data, separating the words, and deactivating the data.

In addition to the preprocessing operation performed on the microblog text dataset, the Word2Vec tool must be used for the word vector training in this particular paper. Because the Skip-gram model is constantly adjusted to the target words based on the prediction of the context, we use it in our research to train and obtain the word vector files. Although the number of predictions is higher, the learning effect is also significantly more beneficial.  Table 1 presents the training process's various parameters in an illustrated format.

### 4.3. Evaluating Indicators

**Figure 6:**
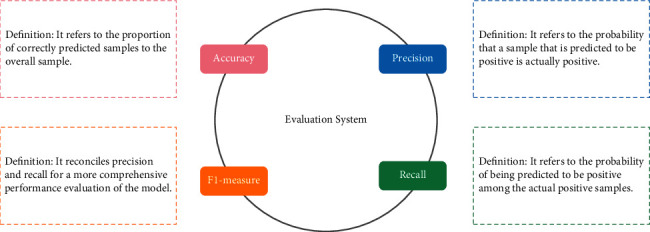
Evaluation index system.

Common evaluation indicators for sentiment classification include accuracy, precision, recall, and *F*1-measure. Sentiment classification is a form of text classification. Because of this, the purpose of this paper is to evaluate the effect of the experimental model for sentiment classification using the aforementioned four common evaluation criteria, and Figure 6 provides an illustration of the detailed evaluation index system.

### 4.4. Results and Analysis

#### 4.4.1. Validation of OCC Sentiment Annotation

The natural labeled dataset was used as the comparison dataset and as input to the Bi-LSTM sentiment recognition model for performance testing respectively. The experimental results of the comparison are illustrated in Table 2, and the purpose of this was to verify the effect that the OCC sentiment rule labeled dataset had on the effect of the sentiment recognition model.

The experimental comparison results are presented in Table 2, and they demonstrate that when compared with the manual natural annotation method, the implementation of the OCC sentiment rule system for the sentiment annotation of college online opinion datasets results in a significant increase in the effectiveness of the model. It is possible that this is because many college and university students do not use emotion words when expressing their feelings on social network platforms like microblogs. This is something that is common among students at those institutions. For this situation, natural annotation methods often cannot determine the sentiment attributes of these texts, so the annotators may give annotation results based on the prevailing environment or mood. However, this results in less standardized sentiment annotation and eventually affects the recognition performance of the model. In order to solve issues of this nature, the OCC has developed sentiment rules that provide a rational and standardized annotation system. Because of this, the recognition performance of the model is improved, and it also explains the reasons for affective cognition from the perspective of cognitive psychology. Additionally, it makes the sentiment labeling of university online opinion datasets more accurate and reasonable.

#### 4.4.2. Comparison Experiments with SVM, CNN, and LSTM Evaluation Models

**Figure 7:**
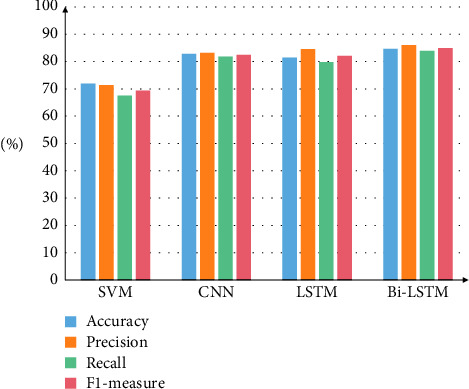
Emotion recognition results of different methods.

Other comparative experiments are designed in this paper with the intention of completely confirming the model's efficacy in the sentiment recognition of university online public opinion. On the university public opinion dataset that was constructed for the study, comparison experiments are carried out utilizing a Bi-LSTM model in conjunction with SVM, CNN, and LSTM models.  Figure 7 provides a visual representation of the findings from the experiments.

The overall classification performance of the neural network methods is significantly better than that of the SVM method when using the same sentiment labeled dataset and the same word vector training, as shown in Figure 7. This is in comparison to the traditional machine learning method, which is illustrated in the figure. Among these models, the Bi-LSTM-based sentiment classification model achieves more satisfactory classification results in all four evaluation indicators, and its overall classification performance is significantly better than that of the SVM, CNN, and LSTM models. Although the LSTM model has long sequence processing capability, the experimental results show that the accuracy of the CNN model is slightly higher than that of the LSTM model. In addition, in comparison to the LSTM model, the Bi-LSTM algorithm is able to obtain feature sequences containing textual contextual information when processing text data. These feature sequences contain both their own unique information as well as the correlation information of the whole text data, which enhances the discriminability, resulting in more satisfactory classification results.

## 5. Conclusion

As a result of the Internet's ever-increasing popularity in today's society, a substantially greater number of individuals take part in online conversations. College students are willing to express their feelings, elaborate on their positions, and reflect their demands through the use of social platforms because they are active participants in the online platform. Because of this, relying on the textual data in university online public opinion and utilizing appropriate evaluation models to analyze the sentiment tendencies of college students hidden in these data can help universities grasp the ideological dynamics and mental health status of college students in a timely manner, discover some existing problems and prevent them before they happen, and provide better assistance to schools and student management as much as possible. In this paper, with the assistance of relevant data that was crawled from the public platform of college microblogs, we develop a sentiment analysis model for college students on the basis of the OCC model and the Bi-LSTM neural network from the perspective of affective cognition and deep learning. The goal of this model is to identify college students' sentiment tendency in university online public opinion. The comparative results show that the model for sentiment recognition that was constructed during the research has achieved ideal results in sentiment recognition of college students, and it has certain feasibility. This is demonstrated by the fact that the model was able to achieve these ideal results.

According to the findings presented above, the research on the sentiment recognition of college students conducted by this study has yielded satisfactory results, and the performance of the sentiment recognition model has been significantly improved. Having said that, there are a few areas that could use some additional research and development: (1) in terms of data collection, this study only collected data from the public microblogging platforms of colleges and universities. And the data sources after preprocessing are not rich enough in terms of data volume and data dimension although they have higher credibility. In the future, it is necessary to further expand the data sources and improve the comprehensiveness of the data. (2) Meanwhile, this study only conducts sentiment analysis research on text data in social platforms. In the follow-up, we can explore the sentiment recognition method of multi-source data that combines image and text data, and find a model with stronger generalization ability to better realize the sentiment classification of college students. (3) At last, based on the sentiment classification model of college students, this paper only studies the problem of positive, negative and neutral three classifications. And people's emotions are rich and diverse, so it is hoped that a more fine-grained multisentiment recognition model can be studied in the future.

## Figures and Tables

**Table 1:** Parameter settings in the Skip-gram model.

Parameters	Values
Window size	5
Minimum word frequency	5
The dimension of output word vector	300

**Table 2:** Effect of different sentiment labeling methods on sentiment recognition results.

Sentiment labeling methods	Accuracy	Precision	Recall	*F*1-measure
OCC sentiment annotation	86.27	84.02	85.67	84.84
Artificial sentiment annotation	78.16	76.24	76.51	76.38

## Data Availability

The labeled dataset used to support the findings of this study can be obtained from the corresponding author upon request.
